# HEALTH LITERACY AMONG OLDER ADULTS IN SWITZERLAND: CROSS-SECTIONAL EVIDENCE

**DOI:** 10.1093/geroni/igac059.609

**Published:** 2022-12-20

**Authors:** Clément Meier, Sarah Vilpert, Carmen Borrat-Besson, Gian Domenico Borasio, Ralf J Jox, Jürgen Maurer

**Affiliations:** University of Lausanne, Lausanne, Vaud, Switzerland; University of Lausanne, Lausanne, Vaud, Switzerland; University of Lausanne, Lausanne, Vaud, Switzerland; Lausanne University Hospital and University of Lausanne, Lausanne, Vaud, Switzerland; Lausanne University Hospital and University of Lausanne, Lausanne, Vaud, Switzerland; University of Lausanne, Lausanne, Vaud, Switzerland

## Abstract

Despite being widely regarded as a major cause of health inequalities, little is known regarding levels of health literacy among older adults in Switzerland. To fill this gap, this study assesses health literacy and its associations with individuals’ social, regional, and health characteristics in a nationally representative sample of adults aged 58 years and older in Switzerland. We use data of 1’625 respondents from a paper-and-pencil self-completion questionnaire that was administered as part of wave 8 (2019/2020) of SHARE in Switzerland. Health literacy is measured using the short version of the European Health Literacy Survey questionnaire (HLS-EU-Q16). We use multivariable regressions to explore how respondents' sociodemographic characteristics are independently associated with health literacy. Overall, 6,8% of the respondents had inadequate health literacy, 24,6% problematic health literacy, and 68,6% sufficient health literacy. There were significant associations between health literacy and individuals' gender, education, economic situation, and self-rated health. Women had higher levels of health literacy than men (p < 0,001). Moreover, a higher education level (p < 0,001), fewer financial difficulties (p< 0.01), and higher self-rated health (p < 0,001) were positively correlated with adequate/higher levels of health literacy. One-third of older citizens have difficulties managing health-related issues in Switzerland. These findings call for targeted interventions, such as using simplified health or eHealth information tools, improved patient-provider communication, and shared decision-making, promoting lifelong learnings activities and health literacy screening for older patients to increase low health literacy and mitigate its consequences, thereby alleviating remaining social health inequalities in the Swiss population.

